# The associations of lens power with age, axial length and type 2 diabetes mellitus in Chinese adults aged 50 and above

**DOI:** 10.1186/s40662-020-00222-2

**Published:** 2020-12-01

**Authors:** Luyao Ye, Jiangnan He, Xinji Zhang, Yi Xu, Qiuying Chen, Yao Yin, Ying Fan, Lina Lu, Jianfeng Zhu, Haidong Zou, Xun Xu

**Affiliations:** 1 Department of Preventative Ophthalmology, Shanghai Eye Disease Prevention and Treatment Center, Shanghai Eye Hospital, No.380 Kangding Road, Shanghai, 200040 China; 2 Department of Ophthalmology, Shanghai General Hospital, Shanghai Jiao Tong University, National Clinical Research Center for Eye Diseases, Shanghai Key Laboratory of Ocular Fundus Diseases, Shanghai Engineering Center for Visual Science and Photomedicine, Shanghai Engineering Center for Precise Diagnosis and Treatment of Eye Diseases, No.100 Haining Road, Shanghai, 200080 China; 3 Department of Health Statistics, Naval Military Medical University, No.800 Xiangyin Road, Shanghai, 200433 China

**Keywords:** Lens power, Type 2 diabetes mellitus, Chinese adults, Cross-sectional study

## Abstract

**Background:**

To investigate the associations of lens power with age, axial length (AL), and Type 2 diabetes mellitus (DM) in Chinese adults aged 50 and above.

**Methods:**

Random clustering sampling was used to identify adults aged 50 years and above in urban regions of Shanghai. The participants underwent a comprehensive ophthalmic examination including subjective refraction, autorefraction, and IOL-Master. The crystalline lens power was calculated using Bennett’s formula.

**Results:**

A total of 4177 adults were included. A linear decrease in lens power was observed both with age and with AL, followed by a stop of lens power loss after the age of 70 or when AL ≥ 25 mm, respectively. Participants with Type 2 DM presented higher lens power (0.43 diopter (D), *p* < 0.001) and thicker lens thickness (0.06 mm, *p* < 0.001). In multivariate regression models, there was a positive correlation between lens power and Type 2 DM when age < 75 years (*p* < 0.001) or AL < 25 mm (*p* < 0.001) after adjusting for other factors, while no significant association was found in participants aged ≥ 75 years (*p* = 0.122) or with AL ≥ 25 mm (*p* = 0.172).

**Conclusions:**

The lens power in adults aged 50 and above exhibited two stages with age and with AL. Type 2 DM caused an increase in lens power, which was not seen in participants aged ≥ 75 years or with AL ≥ 25 mm.

## Background

Refraction is largely determined by corneal power (CP), axial length (AL), and lens power. CP stabilizes in the first year or two after birth [1]. AL presents a natural elongation with aging from newborns but tends to be stable after early adulthood [2]. Apart from that, there is an accelerated increase in AL when myopia develops [3]. Lens power, however, exhibits a lifelong pattern of change. According to the previous studies, the lens power decreases steadily from infancy to early childhood in compensation for AL elongation during the emmetropization process [2], followed by a slower but significant decline at the age of 10–14 [4] and then a near plateau at the age of 14–18 [5]. In adulthood, the lens power exhibits a monotonous decrease with a steeper decline after the age of 55 [6–8] and gains power in adults who develop nuclear cataract [2, 9]. However, most studies only investigated differences in lens power in adults aged < 70 or the proportion of adults aged 70 and above was rather small, therefore, the changes in lens power during this period remain unclear.

The crystalline lens has a biconvex shape, and its curvature is determined by a balance between new fiber production and nuclear compaction [2]. Lens power is composed of the surface power given by its curvatures, and an internal power originating from the profile of its gradient index structure [2]. Any change in these parameters would result in a change in lens power. Hyperglycemia leads to a more convex lens with a lower refractive gradient index through lens hydration [10, 11], which was demonstrated in participants with Type 1 diabetes mellitus (DM) [12–14]. However, the impact of Type 2 DM on lens biometry remains controversial [12, 13, 15]. Since there are ethnic differences in both refraction and Type 2 DM [16, 17] and the existing studies were conducted in the European population with small sample size, the correlation of lens power with Type 2 DM in Asians still remains unknown.

Lens power cannot be measured in vivo but can be obtained either by phakometry or calculated based on other ocular biometry, for example, using Bennett’s formula, which is developed based on the Gullstrand-Emsley model eye [18]; the revised constants [19] and the accurate measurements of biometric parameters ensure the validity of the formula, and thus makes it applicable for large-scale epidemiological research studies.

Therefore, we included a large sample of Chinese adults aged 50 and above with a higher proportion of adults aged ≥ 70 in this cross-sectional study. The lens power was calculated with Bennett’s formula, and its associations with age, AL, and Type 2 DM were explored.

## Methods

### Sampling and enumeration

This cross-sectional study was conducted in adults aged 50 and above in two urban regions of Shanghai. According to the local Chronic Diseases Prevention and Control System, Type 2 diabetic residents diagnosed by doctors from the Beixinjing community, Changning district (an urban region in Shanghai with good management of residents with Type 2 DM) were randomly selected and examined. The sampling procedure was described by He et al. [20]. During August to September in 2016, we conducted the Shanghai Eye Study for Older People (SESOP) survey to screen, identify, and correct blindness and visual impairment among adults aged 50 and above in the Xuhui district (an urban region in Shanghai). Based on the Household Resident Register Record administrated by the district administration, 19 geographically defined clusters (a population of approximately 1000 individuals of all ages) were randomly chosen depending on the percentage of the population that were 50 years and above. Participants were visited individually and written informed consent forms were obtained from them. The exclusion criteria were adopted from the diabetic eye survey; individuals with a history of eye surgery and eye diseases such as glaucoma, corneal opacity, pterygium, eyeball defect and atrophy, retinal detachment, optic nerve atrophy, severe cataract, and amblyopia (best-corrected vision acuity ≤0.5) were excluded. In addition, individuals with Type 2 DM history or abnormal fasting glucose levels (≥ 7.0 mmol/L) were excluded as well as eyes with AL ≥ 30 mm due to possible inaccurate measurement by ocular biometry. This study was approved by the Ethics Committee of Shanghai General Hospital, Shanghai Jiao Tong University (Approval number: 2013KY023 for the diabetic eye survey and 2015KY153 for the SESOP study), in accordance with the tenets laid out by the Declaration of Helsinki.

### Eye examination and calculation

The protocol for the SESOP survey was adopted from the diabetic eye survey using the same examination methods summarized below. Examination sites were set up in each cluster and most of the participants reached the sites by walking within half an hour. Participants were required to bring their official photo identification cards for verification. The fieldwork was conducted by an experienced team of 2 ophthalmologists, 10 optometrists, 4 general health practitioners, and 1 supervisor with sufficient epidemiological study experience. All participants filled out a questionnaire (including age, sex, general and eye disease history, and having DM or not) and underwent a comprehensive eye examination. Subjective refraction was performed by optometrists who achieved the best-corrected vision based on monocular measurement. Spherical equivalents (SE) was obtained as follows: SE = sphere+cylinder/2. Intraocular pressure (IOP) was measured with noncontact tonometry (NT-1000; Nidek, Tokyo, Japan). CP was measured with an autorefractor (Topcon KR 8900; Topcon Corporation, Tokyo, Japan). AL, lens thickness (LT), and anterior chamber depth (ACD) were measured using optical low-coherence reflectometry (Lenstar; Haag-Streit AG, Koeniz, Switzerland). Slit-lamp biomicroscopic examination and fundus examination were performed by experienced ophthalmologists. Fasting (≥ 8 h) venous blood samples were collected and analyzed for blood glucose at Labway Clinical Laboratory Shanghai Co., Ltd. Diagnosis and treatment recommendations were given to participants after the examination. All clinical data were collected, stored in a shared drive and accessed through the local area network by key personnel involved in the study.

Ophthalmologists classified and graded cataract according to the Lens Opacity Classification System (LOCS) II standard color photographs [21]. Participants were classified as “no or early cataract” if nuclear, cortical, and posterior subcapsular lens opacity were LOCS II grade NI, CI, or PSCI or less, respectively. Participants were classified as “advanced cataract” if lens opacity was NII-III, CII-III, or PSCII-III, respectively [22]. Severe cataract that was LOCS II grade equal to or greater than NIV, CIV, and PSCIV were excluded.

Lens power was calculated using Bennett’s formula:

P_L_ = (−1000n(S_cv_ + CP))/(1000n − (ACD + c_1_LT)(S_cv_ + CP)) + 1000n/(−c_2_LT + V). V = AL − LT − ACD is the vitreous body depth, S_cv_ = SE/(1 − 0.014SE) is the spherical equivalent refraction at the corneal vertex, *n* = 4/3 of the aqueous and vitreous indices, *c*_*1*_ = 0.596 and *c*_*2*_ = − 0.358 as estimated using the Gullstrand-Emsley eye model [19].

### Statistical analyses

The variables were presented as proportions for categorical data and as mean ± standard deviation (SD) for continuous data. Student’s t-tests for means or Chi-squared test for proportions (between the control group and the Type 2 DM group) and one-way ANOVA with Bonferroni correction for post hoc tests (for AL categories and age groups) were performed accordingly. Correlations of the ocular biometry were analyzed with Pearson’s correlation coefficients. Multiple regression analysis was performed to explore the association between lens power and Type 2 DM after adjusting for the other factors stratified by the age of 75 and AL of 25 mm, separately. Statistical significance was set at *p* less than 0.05 (2-tailed). Statistical analysis was performed using SPSS v. 22 software (IBM, Armonk, NY, USA). The missing data and data with gross errors were not included in the analyses. Only the right eye was used for analysis since the lens power, AL, and SE of the two eyes were strongly correlated (*r* = 0.513, *r* = 0.740, and *r* = 0.832, respectively; all *p* < 0.001).

## Results

A total of 5501 participants (aged ≥ 50) were examined. 855 right eyes were excluded due to their surgical history (266 eyes), corneal opacity and scar (49 eyes), pterygium (98 eyes), optic atrophy (17 eyes), retinal detachment (5 eyes), glaucoma with significant postoperative complications and vision loss (24 eyes), severe cataract (396 eyes), best-corrected visual acuity of < 20/40 (25 eyes), and AL ≥ 30 mm (120 eyes). Participants with Type 2 DM history or abnormal fasting blood glucose from the SESOP survey (469 eyes) were excluded as well, the age and sex distribution of which were comparable with those from the diabatic eye survey in 2014 (age: 66.37 ± 7.70 and 67.45 ± 8.13, respectively, *p* = 0.001; proportion for men: 44.62 and 42.91%, respectively, *p* = 0.380). Finally, a total of 4057 eyes were included in this analysis.

### General characteristics

Table 1 presents the general characteristics of participants split into having Type 2 DM or not. The mean age, lens power, LT, AL, and SE of participants without Type 2 DM were 65.26 ± 7.92 years, 21.18 ± 2.07 D, 4.63 ± 0.32 mm, 23.61 ± 1.27 mm, and −0.04 ± 2.60 D, respectively. Participants with Type 2 DM were older (Student’s t-test, *p* < 0.001), exhibited significantly higher IOP (*p* < 0.001), higher lens power (*p* < 0.001), thicker LT (*p* < 0.001), and higher CP (*p* = 0.010), and had higher proportions of men and advanced cataract (Chi-squared test, both *p* < 0.001) than those without Type 2 DM, while no significant difference was observed in height, AL, SE, and ACD between two groups (*p* = 0.070 to 0.999, Table 1).

**Table 1:** General characteristics of participants with and without Type 2 DM

Characteristics	Control group		Type 2 DM group		*p* value*
	N	Mean ± SD or %	N	Mean ± SD or %	
Age (years)	2118	65.26 ± 7.92	1939	67.08 ± 7.90	< 0.001
Sex (male)	776	36.64	841	43.37	< 0.001
Height (cm)	2101	162.26 ± 7.98	1917	162.26 ± 8.39	0.999
Lens power (D)	1887	21.18 ± 2.07	1883	21.61 ± 2.17	< 0.001
LT (mm)	1928	4.63 ± 0.32	1912	4.69 ± 0.36	< 0.001
AL (mm)	2118	23.61 ± 1.27	1939	23.56 ± 1.30	0.202
SE (D)	2118	−0.04 ± 2.60	1939	−0.18 ± 2.75	0.101
ACD (mm)	2068	2.96 ± 0.37	1923	2.99 ± 0.36	0.070
CP (D)	2116	44.10 ± 1.47	1921	44.22 ± 1.47	0.010
IOP (mmHg)	2104	13.81 ± 3.00	1882	14.57 ± 2.92	< 0.001
Advanced cataract	331	15.63	332	17.12	< 0.001

Statistical significance was tested using Student’s t-test. Missing data and data with gross errors were not included in the analysis

*ACD* = anterior chamber depth; *AL* = axial length; *CP* = corneal power; *D* = diopter; *DM* = diabetes mellitus; *LT* = lens thickness; *IOP* = intraocular pressure; *SD* = standard deviation; *SE* = spherical equivalents

### Influence of age

**Fig. 1:**
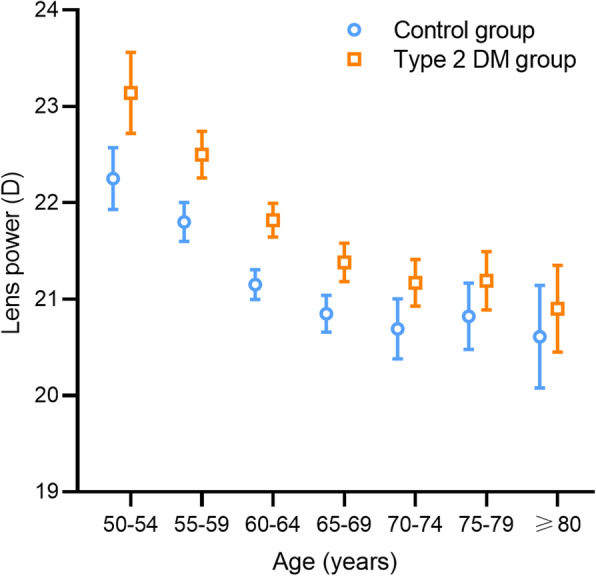
The mean lens power stratified by age for participants with and without Type 2 diabetes mellitus (*N* = 3770). Error bars represent 95% confidence intervals [CI]

**Fig. 2:**
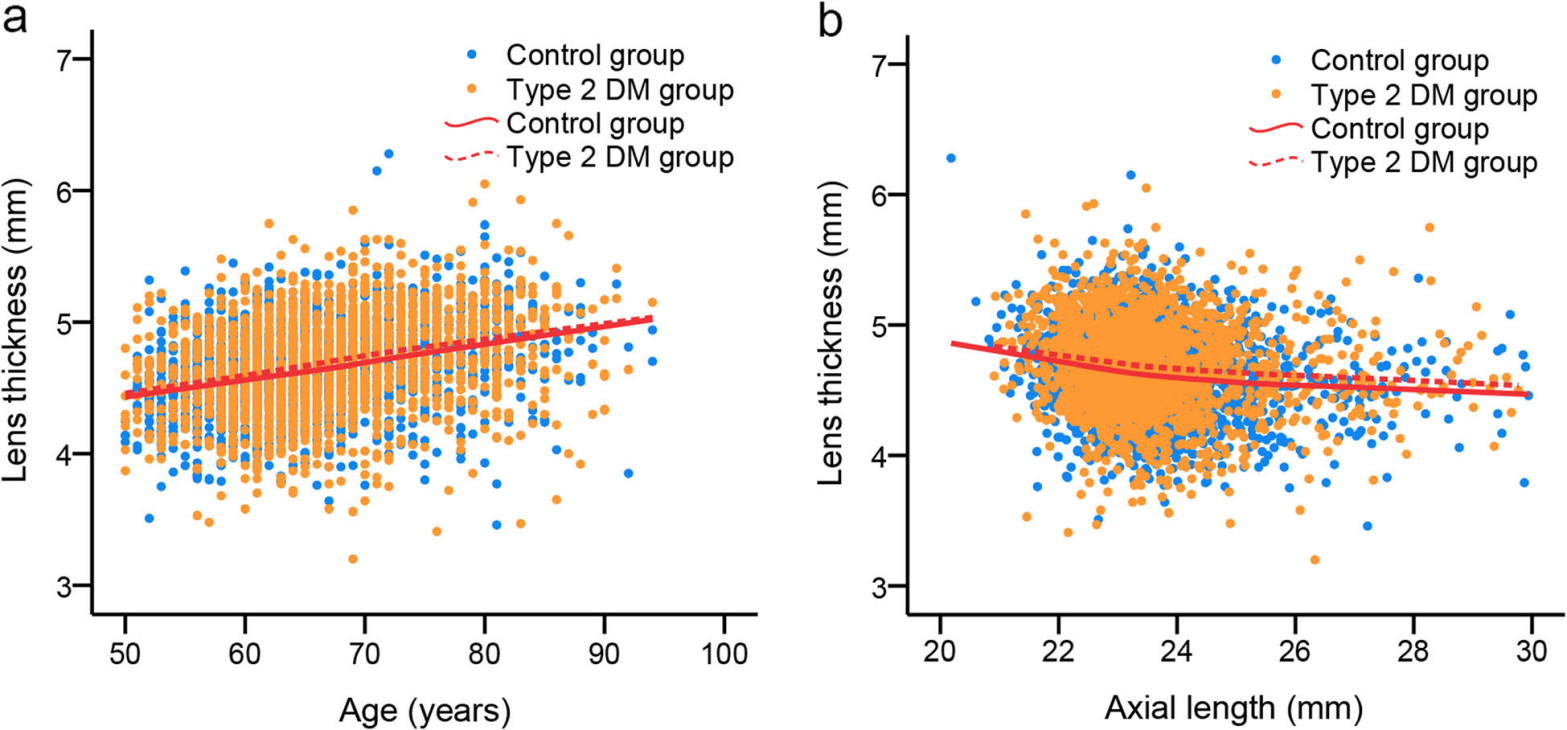
Relationship of lens thickness with age and axial length graphed using linear smoothed (Lowess) plots. **a** Relationship of lens thickness with age and **b** relationship of lens thickness with axial length stratified by having Type 2 diabetes mellitus (DM) or not (*N* = 3840)

In the control group, lens power was negatively correlated with age using Pearson’s correlation analysis (*r* = −0.212, *p* < 0.001) and univariate regression analysis (*p* for trend < 0.001, Table 2). Furthermore, lens power underwent a significantly near-monotonous decrease with age (binned per 5 years) over the elderhood aged 50 to 70 (mean difference of −0.47 D per 5 years, one-way ANOVA with Bonferroni correction, *p* < 0.05), although the difference between age groups of 50–54 and 55–59 was insignificant (*p* = 0.252). However, the difference came to a nearly plateau in participants aged 70 and above (mean difference of −0.04 D per 5 years; both *p* = 1.000; Table 2 and Fig. 1). Besides, a positive association of LT with age was observed (*r* = 0.304, *p* < 0.001; Fig. 2a).

**Table 2:** Distribution of lens power stratified by age for participants with and without Type 2 DM

Age (years)	Control group			Type 2 DM group			*p* value^†^
	N	Lens power (D)	*p* value*	N	Lens power (D)	*p* value*	
50–54	119	22.25 ± 1.78		75	23.15 ± 1.83		0.001
55–59	349	21.89 ± 1.90	0.252	220	22.50 ± 1.83	0.502	< 0.001
60–64	522	21.15 ± 1.81	< 0.001	485	21.82 ± 1.99	0.001	< 0.001
65–69	441	20.85 ± 2.04	0.037	475	21.38 ± 2.21	0.028	< 0.001
70–74	198	20.69 ± 2.21	1.000	296	21.17 ± 2.11	1.000	0.001
75–79	167	20.82 ± 2.26	1.000	209	21.19 ± 2.21	1.000	0.076
≥ 80	91	20.61 ± 2.55	1.000	123	20.90 ± 2.52	1.000	0.418
*p* for trend		< 0.001			< 0.001		

The data was described as mean ± standard deviation

*D* = diopter; *DM* = diabetic mellitus

*p* for trend was measured using univariate regression analysis

* Comparisons with the previous age group using one-way ANOVA with Bonferroni correction

^†^ Comparisons between the control group and the Type 2 DM group for each age group using Student’s t-test

### Influence of AL

**Fig. 3:**
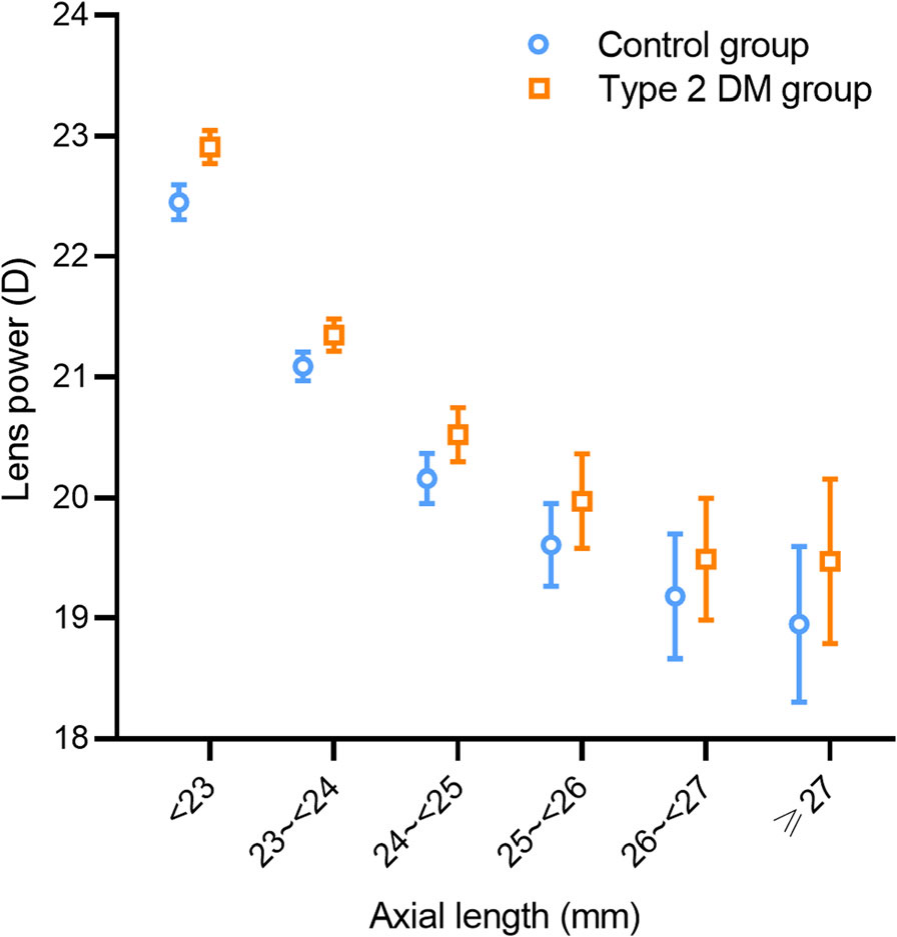
The mean lens power stratified by axial length (AL) for participants with and without Type 2 DM (*N* = 3770). Error bars represent 95% confidence intervals [CI]

There was a negative correlation between lens power and AL in participants without Type 2 DM (Pearson’s correlation analysis: *r* = −0.501, *p* < 0.001; univariate regression analysis: *p* for trend < 0.001, Table 3). When the AL was divided into 1 mm bins, there was a linearly negative correlation between lens power and AL only when AL < 25 mm (mean difference of −1.15 D, one-way ANOVA with Bonferroni correction, both *p* < 0.001), followed by no significant difference when AL ≥ 25 mm (mean difference of −0.33 D, all *p* > 0.05, Table 3 and Fig. 3). There was a negative association between LT and AL (*r* = −0.168, *p* < 0.001). LT showed a declining trend with longer AL and then stabilized at AL of 24–25 mm (Fig. 2b).

**Table 3:** Distribution of lens power stratified by AL for participants with and without Type 2 DM

AL (mm)	Control group			Type 2 DM group			*p* value^†^
	N	Lens power (D)	*p* value*	N	Lens power (D)	*p* value*	
< 23	613	22.45 ± 1.82		688	22.91 ± 1.83		< 0.001
23 ~ < 24	746	21.09 ± 1.67	< 0.001	688	21.35 ± 1.83	< 0.001	0.006
24 ~ < 25	317	20.16 ± 1.88	< 0.001	305	20.54 ± 2.01	< 0.001	0.016
25 ~ < 26	119	19.61 ± 1.90	0.071	100	19.97 ± 1.98	0.158	0.173
26 ~ < 27	45	19.18 ± 1.73	1.000	49	19.49 ± 1.77	1.000	0.391
≥ 27	47	18.95 ± 2.20	1.000	53	19.47 ± 2.49	1.000	0.272
*p* for trend		< 0.001			< 0.001		

The data was described as mean ± standard deviation

*AL* = axial length; *D* = diopter; *DM* = diabetic mellitus

*p* for trend was measured using univariate regression analysis

* Comparisons with the previous AL category using one-way ANOVA with Bonferroni correction

^†^ Comparisons between the control group and the Type 2 DM group for each AL category using Student’s t-test

### Influence of type 2 DM

In the Type 2 DM group, similar patterns of association between lens power and age or AL were observed as seen in the control group (Table 2 and 3, Figs. 1 and 2). Participants with Type 2 DM exhibited significantly higher lens power than the control group when the age < 75 years or the AL < 25 mm (Student’s t-test, all *p* < 0.01). However, the difference in lens power between the two groups disappeared when the age ≥ 75 years or the AL ≥ 25 mm (*p* = 0.076 to 0.418, Tables 2 and 3). Furthermore, multiple regression analysis was performed in subgroups stratified by the age of 75 years and the AL of 25 mm (Table 4). Lens power revealed an independent positive correlation with having Type 2 DM when the age < 75 years (B = 0.591, *p* < 0.001) or the AL < 25 mm (B = 0.506, *p* < 0.001) after adjusting for age, sex, AL, LT, ACD, CP, height, IOP, advanced cataract. However, no significant correlation was found in ≥ 75-year-old group (*p* = 0.122) or ≥ 25 mm AL category (*p* = 0.172).

**Table 4:** Multivariate regression analysis of associations with lens power stratified by age of 75 years and AL of 25 mm

Variables	Age (years)				AL (mm)			
	50 ≤ Age < 75, *N* = 3180		Age ≥ 75, *N* = 590		AL < 25, *N* = 3350		AL ≥ 25, *N* = 420	
	Estimate, 95% CI	*p* value	Estimate, 95% CI	*p* value	Estimate, 95% CI	*p* value	Estimate, 95% CI	*p* value
Age	−0.103 (−0.114, −0.092)	< 0.001	−0.056 (−0.105, −0.008)	0.023	−0.080 (−0.088, −0.071)	< 0.001	−0.077 (−0.107, −0.046)	< 0.001
Sex	0.224 (0.061, 0.387)	0.007	0.027 (−0.497, 0.552)	0.918	0.124 (−0.036, 0.284)	0.127	0.312 (−0.207, 0.830)	0.238
Type 2 DM	0.591 (0.477, 0.705)	< 0.001	0.286 (−0.076, 0.647)	0.122	0.506 (0.395, 0.617)	< 0.001	0.260 (−0.114, 0.635)	0.172
AL	−0.722 (−0.778, −0.666)	< 0.001	−0.982 (−1.216, −0.748)	< 0.001	−1.487 (−1.585, −1.389)	< 0.001	−0.225 (−0.388, −0.062)	0.007
LT	1.132 (0.934, 1.331)	< 0.001	1.153 (0.592, 1.715)	< 0.001	1.133 (0.943, 1. 322)	< 0.001	1.205 (0.571, 1.839)	< 0.001
ACD	−0.623 (−0.843, −0.403)	< 0.001	0.017 (−0.681, −0.715)	0.962	0.094 (−0.132, 0.319)	0.415	−1.113 (−1.782, −0.444)	0.001
CP	−0.195 (−0.238, −0.152)	< 0.001	−0.272 (−0.408, −0.136)	< 0.001	−0.409 (−0.456, −0.362)	< 0.001	−1.133 (−0.260, −0.006)	0.040
Height	−0.023 (−0.033, −0.013)	< 0.001	−0.022 (−0.051, 0.008)	0.151	−0.019 (−0.028, −0.009)	< 0.001	−0.019 (−0.049, 0.011)	0.212
IOP	0.026 (0.007, 0.045)	0.008	−0.032 (−0.091, 0.027)	0.289	0.012 (−0.006, 0.030)	0.201	0.008 (−0.058, 0.074)	0.819
Advanced cataract	0.210 (0.012, 0.407)	0.037	0.535 (0.164, 0.907)	0.005	0.401 (0.232, 0.571)	< 0.001	0.505 (0.001, 1.009)	0.050

Multivariate regression analysis was used to explore the relationship between lens power and Type 2 DM after adjusting for age, sex, AL, LT, ACD, CP, height, IOP, and advanced cataract in subgroups stratified by age of 75 years and AL of 25 mm. R^2^ for age < 75 years: 0.435; R^2^ for age ≥ 75 years: 0.244; R^2^ for AL < 25 mm: 0.408; R^2^ for AL ≥ 25 mm: 0.182

*ACD* = anterior chamber depth; *AL* = axial length; *CI* = confidence intervals; *CP* = corneal power; *D* = diopter; *DM* = diabetic mellitus; *IOP* = intraocular pressure; *LT* = lens thickness; *SD* = standard deviation; *SE* = spherical equivalents

## Discussion

The present study was a cross-sectional study exploring the distribution of lens power and its association with age, AL, and Type 2 DM in Chinese adults aged 50 and above, including a higher proportion of adults aged ≥ 70 years (29.20%) than previous studies. The results revealed a linear decrease in lens power with age and with AL, followed by a stop of lens power loss over the age of 70 or when AL ≥ 25 mm, respectively. Additionally, participants with Type 2 DM caused an increase in lens power, which was affected by older age and longer AL.

The mean lens power in the control group was in agreement with the value obtained by He et al. [8] (20.34 ± 2.24 D), who calculated the lens power with modified Bennett-Rabbetts formula, but was found to be less than another population-based study (24.96 ± 2.18 D) [6]. The differences can be attributed to the younger age of the participants in the latter study (mean age of 44.2 ± 14.2 years), different formula for lens power calculation (modified Bennett-Rabbetts formula), and different ethnic group (European).

The current study found that the lens power exhibited a monotonous decrease in adults aged 50–70 years, followed by no correlation with age over 70 years of age. The insignificant difference in lens power between the age groups of 50–54 and 55–59 was probably due to the small sample size for the age group of 50–54. The pattern in the first stage was consistent with previous studies. Atchison et al. [23] found a decrease in lens power with age (18–69 years) measured by phakometry, which was supported by Jongenelen et al. [6] and Iribarren et al. [7], who reported a decrease in lens power in participants aged 50 and above with modified Bennett-Rabbetts formula and Bennett’s formula, respectively. However, because these previous studies only investigated differences in lens power in adults aged < 70 or that the proportion of adults aged 70 and above was rather small, the distribution of lens power in adults aged ≥ 70 years was unclear. Our study had a higher proportion of adults aged ≥ 70 years (29.20%) and our data indicated that the difference in lens power came to a near plateau with a continuous increase in LT in this age group. The growing aging lens naturally lost power by nuclear compaction and thus an abrupt climbing gradient profile, which may have outpaced the increase in surface lens power originating from a possible greater increase in axial thickness than that in equatorial diameter [2]. However, the lens power loss would reach a relative endpoint as a central gradient plateau was formed and nuclear fibers reached a maximal compaction [10], which might explain the stop of lens power loss after the age of 70. On the other hand, there is a myopic shift in the elderly who develop severe nuclear cataract by altering the refractive index of the lens [9]. Therefore, the gained lens power originating from advanced nuclear cataract might compensate for the natural lens power loss, and thus leading to the plateau of lens power.

We found that lens power exhibited two stages with AL in adults aged 50 and above. A linear decrease in lens power with AL was noted in eyes shorter than 25 mm, followed by no significant difference in eyes with longer AL. A similar pattern of association was observed in LT with AL. The thinner lens axial thickness and loss of lens power in compensation for AL elongation in the first stage were consistent with the previous studies [4–6, 8, 24]. The internal mechanism might lie in a lower growth rate of the lens epithelial layer mediated by fibroblast growth factor from the retina with AL elongation, leading to a thinner LT and surface power loss as well as a more abrupt climbing gradient profile since the rate of compaction could be constant [2]. However, the ability of compensation was limited when approaching a relatively maximal abrupt climbing profile, which might explain the pattern of lens power with AL in the second stage. Cheng et al. also found a limited compensation ability of lens power for AL elongation in highly myopic children and adolescents [24].

Previous studies reported thicker axial lens thickness (0.20–0.37 mm) and lower refractive index of the lens in participants with Type 1 DM compared with the control group [12–14] while the impact of Type 2 DM on lens biometry remained controversial [12, 13, 15]. Wiemer et al. [13] reported no significant difference in lens biometry between participants with Type 2 DM and the control group. However, the current study found thicker LT (0.06 mm) and higher lens power (0.43 D) in Chinese elderly with Type 2 DM, which were consistent with the results reported by Sparrow et al. [15] that revealed a moderate impact of late-onset diabetes on axial lens thickness (0.12 mm), especially on cortex thickness (0.11 mm) with Scheimpflug. Lens hydration was the most prevailing hypothesis that explained the change of lens dimension in participants with DM [10, 11], which was strongly supported by the generalized growth of lens in DM found by Wiember et al. [12] With sorbitol dehydrogenase, excessive glucose absorbed by lens due to hyperglycemia is converted into sorbitol and fructose (polyol pathway), which has poorer permeability through lens membrane. This in turn causes an osmotic pressure gradient between lens and aqueous humor, followed by water influx and lens enlargement [25].

The current study further revealed that the association between lens power and Type 2 DM disappeared in participants older than 75 years or with AL ≥ 25 mm, indicating that the influence of hyperglycemia on lens was affected by age and AL. This might be due to the thorough compaction of lens fibers in participants with older age and the limited number of fibers in response to the hyperglycemia caused by the low growth rate of the lens epithelial layer as AL elongates [2].

The study has strengths of a large sample size of participants aged 50 and above with a higher proportion of participants aged 70 and above than the previous studies. However, the potential limitations of our study should be mentioned. First, even though the distribution of age, sex, height, and AL of the two selected regions were comparable and the socioeconomic backgrounds were similar, there might be other factors influencing the distribution of ocular biometry. Further studies in a single and large population is needed to adequately characterize lens power and its associated factors. Besides, the nonparticipating population could have led to a selection bias. Second, the lens power was calculated with Bennett’s formula but not measured directly by phakometry (the gold standard) although a good agreement between the two methods had been reported previously [19]. Thirdly, the current study was a cross-sectional study, and thus the causal effects of the associations we observed cannot be determined.

## Conclusions

The results of the study demonstrated that the lens power presented a linearly decreasing trend with age and AL and came to a plateau when age ≥ 70 years and AL ≥ 25 mm. Type 2 DM caused an increase in lens power. The negative association between lens power and Type 2 DM was influenced by age and AL, which was not seen in participants aged ≥ 75 years or with AL ≥ 25 mm.

## Data Availability

The datasets used and/or analyzed during the current study are available from the corresponding author on reasonable request.

## Abbreviations

ACD: Anterior chamber depth
AL: Axial length
CP: Corneal power
D: Diopter
DM: Diabetes mellitus
IOP: Intraocular pressure
LOCS: Lens Opacity Classification System
LT: Lens thickness
SE: Spherical equivalents
SESOP: Shanghai Eye Study for Older People
SD: Standard deviation
